# Evaluation of a commercial genetic test for fescue toxicosis in pregnant Angus beef cattle^1^

**DOI:** 10.1093/tas/txaa181

**Published:** 2020-10-01

**Authors:** Justine M Galliou, Piush Khanal, Kyle Mayberry, Matt H Poore, Daniel H Poole, Nick V L Serão

**Affiliations:** 1 Department of Animal Science, North Carolina State University, Raleigh, NC; 2 Department of Animal Science, Washington State University, Pullman, WA; 3 Department of Animal Science, Iowa State University, Ames, IA

**Keywords:** *Bos taurus*, *Epichloë coenophiala*, fescue toxicity, host genetics, T-Snip test

## Abstract

Most tall fescue [*Lolium arundinaceum* (Schreb.) Darbysh] in the Southeastern United States contains an endophyte that causes fescue toxicosis (FT) in grazing animals, a serious disease that causes approximately $1 billion in economic losses to the animal industries in the United States. Recently, a genetic test called T-Snip (AgBotanica, LCC, Columbia, MO), was developed with the objective of identifying animals with genetic variation for FT tolerance. The aim of this study was to validate the use of this genetic test in mature, pregnant cows. Over 13 wk, weekly phenotypic data, including body weight, rectal temperatures, hair coat scores, hair shedding scores, and body condition scores, were collected on 148 pregnant purebred Angus cows at 2 locations in NC where infected fescue was the primary source of feed. Birth weights (cBW) and 205-d adjusted weaning weights (adjWW) from these cow’s calves were recorded. All cows were genotyped for T-Snip. At the end of the trial, each phenotypic trait was calculated as the slope of the linear regression of performance on weeks. The effect of T-Snip rating genotypes (4 levels) on slope traits was tested using a linear model also including the fixed-effects of location, parity, and the initial measurement for each trait (covariate). For cBW and adjWW, the model also included the sex of the calf and the month of birth as categorical effects. Associations of T-Snip genotypes were observed for body weight gain (aBWd) of pregnant cows (*P* = 0.15; interaction with location), change in body condition score (aBCSd; *P* = 0.13), and adjWW (*P* = 0.06; interaction with location). For aBWd and adjWW, associations were found just within one location (*P* = 0.017 and 0.047, respectively), which was the location with higher endophyte infection rate. For all associations, the direction of the T-Snip genotypes was the same and as expected: the greater the genotype score, the better performance. No associations were found for the other traits (*P* > 0.10). These results indicate that the T-Snip test may be predictive of cow performance (aBWd, aBCSd, and adjWW) in an endophyte-infected tall fescue environment.

## INTRODUCTION

Tall fescue [*Lolium arundinaceum* (Schreb.) Darbysh] is the predominant forage available in the southeastern United States, and most of it contains a fungal endophyte, *Epichloë coenophiala*, which produces ergot alkaloid compounds causing fescue toxicosis (FT) in grazing animals (Young et al., 2013). Tall fescue was introduced to the Southeastern United States in the 1940s, primarily as the variety Kentucky-31 tall fescue. Kentucky-31 tall fescue had excellent agronomic performance, but after rapid adoption across a wide region known as the fescue belt it was recognized that cattle grazing it during hot weather suffered from a toxicosis syndrome which included sensitivity to heat, slow shedding of winter hair coat, reduced milk production, reduced growth rates and weaning weights, and reduced breeding rates (Studemann and Hoveland, 1988). These problems are caused by the ingestion of ergot alkaloids, primarily ergovaline, which was shown to cause reduced prolactin levels in blood and vasoconstriction in the extremities (Aiken and Strickland, 2013), and symptoms are exacerbated by high environmental temperatures (Poole et al., 2019). It was once thought that vasoconstriction was limited to the peripheral circulation, but recently vasoconstriction has been reported in the ruminal (Foote et al., 2011), and uterine and ovarian blood vessels (Poole et al., 2018). Total cost of the various symptoms of FT are generally thought to exceed $1 billion dollars annually (Strickland et al., 2011).

The host genetics of FT are poorly understood. Several studies found differences in response to the ergot alkaloids between breeds (Browning, 2000, 2004; Cole et al., 2001; Burke et al., 2010). However, information about variation within breeds is necessary for genetic improvement to FT. Gray et al. (2011) reported a heritability estimate for hair shedding which provides some insight into the heritability of FT. These authors showed that adaptability score (month of first shedding) was moderately heritable (0.35) and had moderate negative genetic correlation with calf weaning weight (−0.58). On the genomic side, the few reports available in the literature have focused on a candidate gene approach (Looper et al., 2010; Bastin et al., 2014; Campbell et al., 2014).

Recently, a genetic test called T-Snip (AgBotanica, LCC, Columbia, MO; https://www.agbotanica.com/t-snip.aspx) has been developed with the objective of identifying animals with different genetic potential for FT response in cattle. Masiero et al. (2016), in partnership with the developers of the genetic test, found a significant correlation between T-Snip scores of dams and weaning weights of calves, ranging from 0.42 to 0.76 in eight herds. However, information regarding the breeds, season, endophyte infection rates, and location are all factors that impact the severity of FT were not provided in the report. McDonald (2017) also evaluated the association between this test and feeder calves receiving endophyte-positive and negative and obtained an association (*P* = 0.05) between this genetic test and average daily gain of feed calves, out of all traits evaluated. McDonald (2017) further suggested that this test should be used to identify adult animals showing tolerance to FT. Therefore, the objective of this study was to evaluate the T-Snip in pregnant Angus cows as a genetic test for tolerance to FT.

## MATERIALS AND METHODS

The protocol for this experiment was reviewed and approved by the Institutional Animal Care and Use Committee at North Carolina State University (NCSU IACUC#13-093-A; 17-043-A).

### Animals and Forage Quality

A total of 150 multiparous (parities 2 to 4) pregnant purebred Black Angus cows and their calves (*n* = 127) were used. Approximately half of the animals (79 cows and 65 calves) were located at the Upper Piedmont Research Station (UPRS—Reidsville, NC), while the remaining animals (71 cows and 62 calves) were located at the Butner Beef Cattle Field Laboratory (BBCFL—Bahama, NC). To ensure cow performance was not impacted by lactational stress, all calves were weaned from cows prior to the start of the grazing period.

Rotational grazing was used for the cattle at these locations 2 wk at each to have adequate forage management and to insure sufficient forage. Forage samples were collected every 2 wk and the nutrient quality and percentage of fescue in the forage were assessed by the North Carolina Department of Agriculture Forage Laboratory (Raleigh, NC). A subset of forage sample was dried, ground and sent to the University of Missouri Veterinary Medical Diagnostic Laboratory (Columbia, MO) for analysis of ergot alkaloid and ergopeptine concentrations present using HPLC as described by Rottinghaus et al. (1993). Ergovaline concentrations were 185 and 316.7 µg/kg were found at BBCFL and UPRS farms, respectively (Table 1). In addition, in November of 2016 fescue tiller samples were collected to evaluate the infection rate for toxic fescue in the pasture. Fescue tiller samples were rinsed, and shipped on ice the following morning in order to determine pasture infection percentage and the average infection rate during the experimental period (Agrinostics Ltd. Co., Watkinsville, GA). At BBCFL, endophyte infection percentage varies between pastures, and cattle rotationally grazed pastures that varied from 65% to 95% infected. In contrast, the endophyte infection rate at UPRS was equal to or greater than 95% for all pastures.

**Table 1.** Ergot alkaloid concentration^1^ of tall fescue pastures by location^2^

Ergot alkaloids	BBCFL	UPRS
Ergosine	0	166.7
Ergotamine	0	87.5
Ergocornine	0	26.7
Ergocryptine	0	75.0
Ergocristine	0	24.2
Ergovaline	185	316.7
Total	185	696.7

^1^µg/kg.

^2^BBCFL, Butner Beef Cattle Field Laboratory (Bahama, NC); UPRS, Upper Piedmont Research Station (Reidsville, NC).

### Phenotypic Measurements

**Figure 1.**
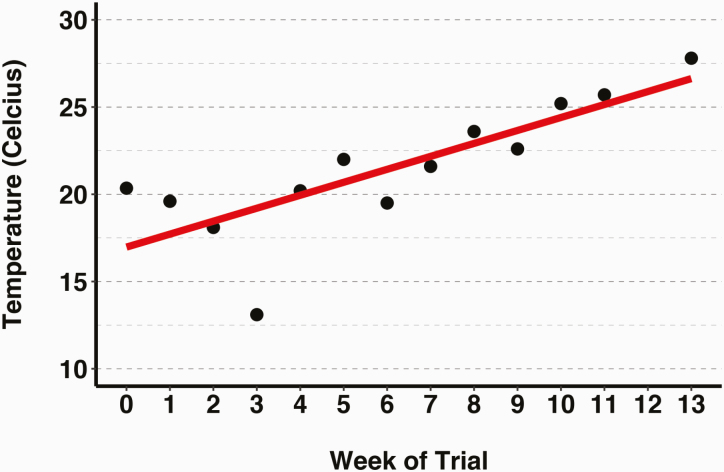
Average temperatures at collection dates. Temperatures ranged from 13.1 °C in May to 27.8 °C in July and showed a positive trend.

Over 13 wk, from April 2016 until July 2016, which is the optimal time to observe the impact of both heat stress on FT, phenotypic data were collected on all cows. The average temperatures during the time of collection ranged from 13.1 °C in May and 27.8 °C in late July. Temperatures steadily increased throughout the 13 wk (Fig. 1) and there were no significant differences between the temperatures at each location with an average of 21.7 °C at Butner and 21.5 °C at UPRS (data not shown).

Weekly phenotypic data collected on each cow included body weight (BW), body condition score (BCS), hair shedding score (HSS), hair coat score (HCS), and rectal temperature (RTemp). In order to reduce bias, two trained evaluators assigned the BCS, HSS, and HCS, and the average score was used for subsequent statistical analyses. Hair shedding scores, ranging from 1 to 5, with 1 being a completely shed out, short summer coat and 5 being a full nonshed winter coat (Gray et al., 2011), whereas HCS, ranging from 1 to 5, with 1 being a short and slick hair coat and 5 being a long dense hair coat (Olson et al., 2003). Blood samples were collected at the start of the study to prepare blood cards for subsequent genotyping with the T-Snip. For the calves, BW (cBW) was measured at birth and at weaning. Calves were born in the fall between the months of September and January. Weaning weight was adjusted for 205 d (adjWW) using the following formula: $adjWW=\frac{weaning\;weight-birth\;\;\;\;weight}{weaning\;age}\times 205$. Calf data were collected following the cow data collection period from late September 2016 through May 2017. The number of steer and heifer calves was 37 and 24 (BBCFL), respectively, and 34 and 32 (UPRS), respectively. One cow died before calving and her data were not used for analyses. In addition, 17 cows did not calve any offspring, 2 calves were born dead, and 3 calves were sold prior to weaning. Thus, the number of calf data was lower than the number of cow data.

### T-Snip Genotyping

**Figure 2.**
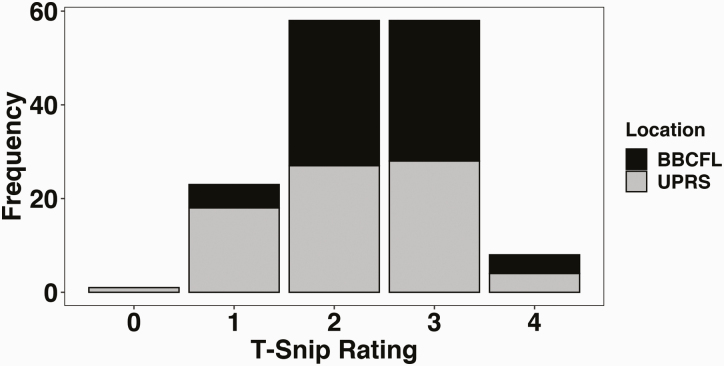
Distribution of T-Snip rating genotypes by location. Genotype values range from “0” to “5”, with greater genotype values association with greater tolerance to FT. Black and gray bars represent the frequency within BBCFL and UPRS, respectively. Cattle in this study displayed T-Snip (AgBotanica, LCC, Columbia, MO) index genotypes values ranging from “0” to “4”, however the single animal with genotype “0” was removed from the overall statistical analyses.

Blood cards were shipped to GeneSeek (Neogen Genomics, Lincoln, NE) for T-Snip genotyping. T-Snip assigns two scores to each animal based on their genotypic variation for tolerance to FT: the tolerance rating, ranging from 0 to 5, and the tolerance index, ranging from 0 to 50, with 0 being least tolerant in both cases. The T-Snip index is generated by analyzing multiple genetic markers; these results are then used to calculate the T-Snip rating. The number of markers used, as well as the specific genes associated with those remain proprietary to AgBotanica, LLC (Columbia, MO). Analyses were performed using both the rating and the index, however, results presented here focus only on associations with the T-Snip ratings. The distribution of the T-Snip ratings are shown in Fig. 2. One animal had problems with genotyping and only one animal had a T-Snip of 0. Both animals were removed from the data, yielding a total of 147 cows with T-Snip genotypes and phenotypes.

### Slope Phenotypes

Individual cow phenotypic measurements recorded across time (BW, BCS, HSS, HCS, and RTemp) were summarized as the slope from the regression analyses of phenotypes on weeks. Slopes were estimated with the objective of assessing the change in individual phenotypic performance as a function of time. A linear regression was used for BW as described by Hassen et al. (2004), for BW is best predicted using a linear regression with a cubic effect of age. While the cubic effect of age was not included in this model, parity was included when testing for associations, thus taking into account differences in age. The estimated slope from each analysis was defined as: average BW difference (aBWd), average BCS difference (aBCSd), average HSS difference (aHSSd), average HCS difference (aHCSd), and average RTemp difference (aRTd). Slopes were estimated based on three time windows: weeks 1 through 13 (w1_13) representing the entirety of the grazing period, weeks 1 through 7 (w1_7), and weeks 7 through 13 (w7_13), in order to assess the potential impact of confounded effects during the study with pregnant cows, such as the increase in temperature and humidity (from April to July), availability and diversity of forages, and chronic exposure to ergot alkaloid in the endophyte-infected tall fescue. Slopes for cBW and adjWW were not estimated as these measurements were taken at one time point only. The summary statistics for the slope phenotypes are presented in Supplementary Table S1.

### Statistical Analyses

The effect of T-Snip rating genotypes on slope traits was tested using the following linear model:

$$\begin{array}{ll} y_{ijklm}= & \;\;\mu +TSnip_{i}+L_{j}+{\left(TSnip*L\right)}_{ij}\\ & +P_{k}+G_{l}+cov_{m}+e_{ijklm} \end{array}$$ (1)

where *y*_*ijk*_ is the estimated slope; *µ* is the overall mean; *TSnip*_*i*_ is the fixed-effect of the *i*th T-Snip rating genotype (l = 1 to 4); *L*_*j*_ is the fixed-effect of the *j*th location; (TSnip*L)_*ij*_ denotes the interaction between *TSnip*_*i*_ and *L*_*j*_; *P*_*k*_ is the fixed-effect of the *k*th parity; *G*_*l*_ is the fixed-effect of the *l*th gestation status (delivered a calf or not); *cov*_*m*_ is the covariate of initial measurement of the *m*th animal (e.g., initial BW for aBWd); and *e*_*ijklm*_ is the random error associated with *y*_*ijklm*_, with $\;e_{ijklm}∼N\left(0,I\sigma_{e}^{2}\right)$, where ***I*** is the identity matrix. Analysis of aRTd included the effect of temperature heat index (THI) as an additional covariate, where this was calculated as the area under the curve of the weekly THI for the period analyzed. The distribution of THI over all 13 wk was normal (data not shown). The effect of week temperature on all other traits evaluated was tested but it was not significant (*P* > 0.05), and thus, removed from final analyses (data not shown). In addition to the effects of *TSnip*, *L*, TSnip*L, and *P*, the statistical analysis of cBW included the fixed-effects of calf sex and calving month, and no covariates, whereas the statistical analysis of adjWW included the fixed-effects of calf sex and calving month, and cBW as the covariate. Normality of the Studentized residuals was evaluated using Shapiro–Wilk’s test and considered normal at *P* > 0.01, and when Studentized residual fell within ±3 standard deviations. Homogeneity of the residuals was assessed visually. Least-squares means of significant effects (*P* < 0.10) were separated using Fisher’s Least Significant Difference test. Trends were considered when *P* < 0.15. A less stringent significance threshold was used in place of the traditional alpha of 0.05 because of the limited number of animals used in this trial (i.e., statistical power), the distribution of genotypes (Fig. 2), and the goal of evaluating a commercial test. In addition, a post hoc orthogonal contrast for the linear effect (i.e., additive) of T-Snip was evaluated because of the results presented by AgBotanica (2018), where there was a clear additive relationship between T-Snip ratings and performance. This contrast was tested for the main effect of T-Snip rating, interaction between T-Snip and location, and within each of the two locations. Analyses were performed is SAS 9.4 (Statistical Analysis System, Cary, NC). Summary statistics of the data after removal of outliers is presented in Table 2.

**Table 2.** Summary statistics used in the final analysis of traits evaluated by location^1^

	BBCFL					UPRS				
Trait^2^	*n*	mean	SD	min	max	*n*	mean	SD	min	max
iBW, kg	70	517.3	62.4	376.0	652.3	77	488.3	59.7	365.6	662.2
fBW, kg	70	539.8	56.8	414.6	664.5	77	490.1	60.7	364.7	666.8
iBCS	70	5.5	0.5	4.25	6.75	76	5.0	0.4	4.0	5.75
fBCS	70	5.5	0.4	4.75	6.50	76	5.3	0.7	4.0	6.5
iHCS	70	4.0	0.5	3.0	5.0	77	3.6	0.8	1.25	5.0
fHCS	70	2.6	0.8	1.25	4.75	77	2.1	1.2	1.0	5.0
iHSS	70	4.2	0.5	3.0	5.0	75	4.0	0.8	1.0	5.0
fHSS	70	3.1	1.0	1.25	5.0	75	2.7	1.1	1.0	5.0
iRTemp, °C	70	38.5	0.5	37.0	39.4	77	39.0	0.6	37.8	40.3
fRTemp, °C	70	38.9	0.4	37.8	40.1	77	38.5	0.4	37.8	39.3
cBW, kg	60	30.8	5.3	11.3	42.2	62	30.3	5.8	15.9	40.8
adjWW, kg	60	229.1	25.3	185.6	301.8	62	234.3	26.5	162	296.4

^1^BBCFL, Butner Beef Cattle Field Laboratory (Bahama, NC); UPRS, Upper Piedmont Research Station (Reidsville, NC).

^2^i, initial; f, final; BW, body weight; BCS, body condition score; HCS, hair coat score; HSS, hair shedding score; RTemp, rectal temperature; cBW, calf birth weight; adjWW, 205-d adjusted calf weaning weight.

## RESULTS

### Distribution of Genotypes

The distribution of T-Snip ratings and rating genotypes are shown in Fig. 2 and Supplementary Fig. S1, respectively. The distribution of ratings by location are shown in Table 3. Ratings of 1 and 2 were uniformly distributed across location, however, UPRS had more animals with ratings of 3 and 4 than BBCFL. The number of cows with T-Snip rating genotypes of 0, 1, 2, 3, and 4 were 1, 22, 59, 56, and 10, respectively. A rating of 5, indicating most tolerant to FT, was as not observed in any of the cows within these two locations. The number of cows by T-Snip index genotypes had an average index of 24 (SD = 8.04), ranging from 5 to 43 (Supplementary Fig. S1).

**Table 3.** Number of animals attributed each T-Snip^1^ rating per location^2^

T-Snip rating	BBCFL	UPRS
0	0	1
1	5	18
2	31	27
3	30	28
4	4	4

^1^AgBotanica, LCC, Columbia, MO.

^2^BBCFL, Butner Beef Cattle Field Laboratory (Bahama, NC); UPRS, Upper Piedmont Research Station (Reidsville, NC).

### Effect of T-Snip Rating

The association between T-Snip rating genotypes by location using data from time windows w1_7 are shown in Table 4. For completeness, results for time windows for w7_13, and for the whole trial (w1_13) are shown in Supplementary Tables S2 and S3, respectively. Overall, results where similar at the different time windows. The major differences will be highlighted below.

**Table 4.** Least-squares means for the effect of T-Snip^1^ (TS) rating genotype (1 to 4) by location (L)^2^

	BBCFL				UPRS					*P-*value		
Trait^3^	1	2	3	4	1	2	3	4	pSEM^4^	TS*L	TS	L
aBWd, kg/w	5.31^a^	3.48^a^	3.98^a^	4.27^a^	0.31^d^	1.20^cd^	1.85^bc^	3.39^ab^	0.62	0.15	0.33	<0.01
aBCSd	0.012^bc^	0.003^c^	−0.001^c^	0.042^abc^	0.031^bc^	0.046^ab^	0.042^ab^	0.107^a^	0.02	0.82	0.13	<0.01
aHCSd	−0.24	−0.20	−0.20	−0.21	−0.21	−0.20	−0.27	−0.31	0.04	0.34	0.49	0.37
aHSSd	−0.20	−0.15	−0.14	−0.15	−0.16	−0.19	−0.25	−0.28	0.04	0.28	0.75	0.09
aRTd, °C	0.09	0.06	0.04	0.01	−0.06	−0.02	−0.08	−0.04	0.03	0.54	0.32	<0.01
cBW, kg	28.2	28.2	25.9	27.8	23.7	23.2	25.4	26.9	1.99	0.18	0.91	0.10
adjWW, kg	226.2^ab^	232.8^a^	216.5^abc^	211.4^abc^	194.3^c^	207.2^bc^	211.1^abc^	226.8^ab^	11.08	0.06	0.48	0.39

^a–d^Least-squares means lacking common superscripts are statistically different (*P*-value <0.1). Post hoc comparisons were added for the traits showing significant (*P*-value <0.1) effect of TS*L, or for contrasts presented in Table 3.

^1^AgBotanica, LCC, Columbia, MO.

^2^BBCFL, Butner Beef Cattle Field Laboratory (Bahama, NC); UPRS, Upper Piedmont Research Station (Reidsville, NC).

^3^aBWd, average body weight difference; aBCSd, average body condition score difference; aHCSd, average hair coat score difference; aHSSd, average hair shedding score difference; aRTd, average rectal temperature difference; cBW, calf birth weight; adjWW, 205-d adjusted calf weaning weight.

^4^Pooled SEM, calculated as the weighted average of SEM at each location–genotype combination.

There was a tendency for the interaction between T-Snip rating genotypes and location for aBWd (*P* = 0.15) and a significant interaction for adjWW (*P* = 0.06). The relationship between T-Snip rating genotypes and aBWd was opposite between locations. In addition, the interaction between the linear effect of T-Snip and location was significant (*P* = 0.053; Table 5) for aBWd. Cows located at BBCFL had statistically similar (*P* > 0.10) aBWd from genotypes 1 (5.31 kg/w) to 4 (4.25 kg/w), whereas at UPRS, cows increased (*P* < 0.1) aBWd from genotype 1 (0.31 kg/w) to 4 (3.39 kg/w). These results are in accordance with the linear effect of T-Snip tested within each location (Table 5), with no association (*P* = 0.599) at BBCFL and strong association (*P* = 0.017) at UPRS. For adjWW, there was an increase (*P* < 0.1) in adjWW at UPRS (T-Snip rating genotype 1 = 194.3 kg vs. 4 = 226.8 kg). Additionally, there was a significant interaction (*P* = 0.033) between the linear test for T-Snip and location, in which the linear effect of T-Snip was significant (*P* = 0.047) at UPRS but not (*P* = 0.276) at BBCFL. There were no other traits examined that demonstrated an effect of interaction between T-Snip rating genotype and location (*P* < 0.15).

**Table 5.** Significance^1^ of the linear^2^ effect of T-Snip^3^ genotype

Trait^4^	Overall	Linear*Location	Linear(BBCFL)	Linear(UPRS)
aBWd	0.258	0.053	0.599	0.017
aBCSd	0.050	0.370	0.482	0.027
aHCSd	0.465	0.187	0.702	0.107
aHSSd	0.587	0.129	0.521	0.107
aRTd	0.347	0.428	0.262	0.907
cBW	0.599	0.334	0.768	0.262
adjWW	0.587	0.033	0.276	0.047

^1^Values in the table represent *P*-values.

^2^Overall, contrast representing the linear effect of the main effect of T-Snip; Linear*Location, contrast representing the interaction between the linear effect of T-Snip and location; Linear(BBCFL), contrast representing the linear effect of T-Snip within Butner Beef Cattle Field Laboratory (BBCFL, Bahama, NC); Linear(UPRS), contrast representing the linear effect of T-Snip within Upper Piedmont Research Station (UPRS, Reidsville, NC).

^3^AgBotanica, LCC, Columbia, MO.

^4^aBWd, average body weight difference; aBCSd, average body condition score difference; aHCSd, average hair coat score difference; aHSSd, average hair shedding score difference; aRTd, average rectal temperature difference; cBW, calf birth weight; adjWW, 205-d adjusted calf weaning weight.

There was a tendency of T-Snip rating genotype for aBCSd (*P* = 0.13; Table 4) only. In fact, there was a significant linear effect of T-Snip genotype on this trait (*P* = 0.05; Table 5), with this effect being driven by the association (Table 5) at UPRS (*P* = 0.027) and not at BBCFL (*P* = 0.482). In general, animals with genotype 4 (0.07) had greater (*P* ≤ 0.042) aBCSd than those with genotypes 1, 2, and 3 (0.02 for each). At UPRS, this association was stronger (*P* = 0.027), and similarly, genotype 4 (0.107) had greater aBCSd than all other genotypes. There was also a significant effect of T-Snip genotype (*P* = 0.032, Supplementary Table S2) on aBWd for w7_13, where growth increased from genotype 1 (0.697 kg/w) to 4 (2.378 kg/w). There were no other traits with an effect (*P* < 0.15) of T-Snip genotype. This was also the case when we evaluated the linear effect of the T-Snip (*P* < 0.15, Table 5).

Although the focus of this study was not to compare locations, there was an effect of location for aBWd (*P* < 0.001), aBCSd (*P* < 0.001), aHSSd (*P* = 0.09), aRTd (*P* < 0.001), and cBW (*P* = 0.10). In general, animals at BBCFL had greater gain (aBWd), lower changes in body condition (aBCSd) and hair shedding (aHSSd), increased rectal temperature (aRTd), and greater cBW compared with cattle at UPRS.

## DISCUSSION

### Overall Discussion

Current prevention strategies to reduce the effects of FT on beef cattle include replacing toxic tall fescue with novel endophyte-infected varieties, rotating the livestock to nontoxic pastures, interseeding pastures with other forages in order to dilute the ergot alkaloid producing endophyte infection rates, and changing production systems by using crossing heat resistant breeds like Braham and Senepol cows with English breeds such as Angus and Hereford (Browning, 2004; Burke et al., 2010; Whittier, 2013). Despite these options, FT continues to compromise U.S. beef cattle production because of the high cost of these options (Roberts and Andrae, 2004). The T-Snip is a commercial genetic test recently released with the objective of identifying cattle with improved tolerance to FT (AgBotanica, 2018), which could potentially be used to reduce the adverse effects of FT.

This study utilized two groups of cows that grazed endophyte-infected tall fescue pastures during the period of time with elevated ergot alkaloid concentrations and heat stress, which was optimal for inducing FT in these herds. Examining phenotypic traits under these conditions would effectively determine whether T-Snip would be a useful tool for producers who have to raise beef cattle in a fescue prominent environment. Ergovaline concentrations increase from spring throughout the summer (Rogers et al., 2011), with the signs of FT remaining prevalent through the summer, June–July, suggesting an interaction between FT and environmental temperature (Hemken et al., 1981) and cumulative impacts of toxin consumption (Klotz, 2015). Animals fed endophyte-infected fescue only showed symptoms of FT under heat stress conditions (Hemken et al., 1981; Burke et al., 2001).

As reported by Jacobson et al. (1970) weight gain, hair coat, body temperature, and calf birth, and weaning weights are all traits affected by FT. These traits were evaluated as the change in performance across time, which can be interpreted as the animal’s response due to chronic exposure to FT. With the assumption that the T-Snip accurately identifies tolerant animals to FT, it was hypothesized that more tolerant animals would show increased growth performance and body condition (aBWd, cBW and adjWW, and aBCSd, respectively), and decreased body temperature (aBTd), and hair coat length (aHCSd) and shedding (aHSSd) compared with less tolerant animals.

The high level of physical stress on beef cows, particularly the 2-yr olds, puts these cattle at higher risk of being removed from the herd at an earlier age if less tolerant to FT in a fescue predominant environment. Due to the need to retain these females of high genetic value in a herd, this determining the validity of the T-Snip genetic tests in this population of cattle is of high value to producers. The herds used in this study have been historically selected for overall performance under FT, as signs of FT as well as high endophyte infection rates in the pastures have been extensively utilized throughout the years. Therefore, this group of animals has already undergone some selection based on FT performance, which reduces the wide variety of unknown factors that come with using an unselected group of heifers or growing steers, such as dystocia or susceptibility to disease. Nevertheless, the T-Snip genotypes of these animals remain normally distributed, this may indicate that there was still enough genetic variation for the test to distinguish between tolerant and susceptible animals. Finally, focusing on pregnant animals which provided the ability to observe the impact of FT during pregnancy performance without impacting the potential relationship between the dams’ T-Snip scores and their calves’ performance.

The results from this study showed associations between T-Snip ratings and performance within one location, suggesting the presence of genotype-by-environment interaction. We observed weak associations between T-Snip and performance in the environment with lower percentage of toxic fescue (i.e., BBCFL), whereas much stronger associations were found at the other location (UPRS), which had greater amount of endophyte-infected fescue. While a defined control group (i.e., cattle consuming nontoxic fescue) was not included in the experimental design of this study, another study was conducted at the same time and location with mature multiparous Black Angus cows consuming novel or toxic endophyte-infected tall fescue (Newsome, 2018), provides additional support that cattle reported in this study experienced the symptoms of FT. Hence, the evaluation of a genetic test for FT tolerance must be performed in a toxic-fescue environment. Overall, our results showed that not only the presence of toxic fescue, but also the level of endophyte infection (i.e., toxicity) impacts the results of this test.

Results for w1_7 were presented in the main text due to preliminary analysis using these data (Koester et al., 2020) showed that the estimated additive genetic (2.22 [kg/wk]^2^) and residual (1.09 [kg/wk]^2^) variances during this period were greater than for the other periods, with 1.04 (kg/wk)^2^ and 1.94 (kg/wk)^2^, respectively, for w7_13, and 0.58 (kg/wk)^2^ and 1.09 (kg/wk)^2^, respectively, for 1_13, while having similar heritability estimate for these three time windows (~0.35). This is in accordance to reports showing that, under stress/disease conditions, traits show greater additive genetic and residual variances compared with the same traits under a “clean” environment (Scanlan et al., 2019). Moreover, the correlation between data from w1_7 and w1_13 was higher than for data between w7_13 with w1_7 and w1_13. Altogether, by using the first 7 wk of data, the greatest genetic variation was observed without affecting the overall chronic response to FT.

### Effect of T-Snip Rating on Cow Performance

In contrast to previous reports (AgBotanica, 2018), animals with the greatest weight gain on endophyte-infected tall fescue pastures did not have high T-Snip ratings. Nonetheless, there was a trending significance for the effect of T-Snip by location interaction. Furthermore, this indicated a clear genotype-by-environment effect on this trait, in which aBWd means were the same within BBCFL, but different at UPRS, which had a greater endophyte infection percentage during the grazing period. The additional post hoc linear contrast test supported this, indicating an additive effect of T-Snip ratings, where greater growth was observed as ratings increased. However, there was an overall association between T-Snip rating genotypes when this trait was analyzed during the later period of the trial, which could be due a different dynamic of chronic exposure to toxic fescue at BBCFL not captured during the first 7 wk of the trial. As previously mentioned, there were no differences in temperatures between locations, thus a difference in heat stress specifically at BBCFL during the later period of the trial would not be the cause of this contrast between locations. These results indicate that the T-Snip test is associated with growth in pregnant cows during the summer months, and that a greater endophyte infection percentage in fescue forages available to the animals can improve this association.

The BCS attributed to the animals in this trial ranged from 4.25 to 6.75. Ideal BCS for cattle has been determined to be between 5 and 7 (Eversole et al., 2009). There was a trending association of T-Snip rating genotype with aBCSd indicating that animals with higher genotype ratings were given higher BCS. However, differences in aBCSd occurred at UPRS only, which was further supported by the post hoc linear contrast tested. This result is supported by the results for aBWd, in which greater T-Snip rating genotypes were observed in animals that grew faster during the first 7 wk of the trial. Similarly, it seems that the exposure to greater endophyte infection percentages may impact the association between T-Snip rating genotypes and this trait.

As for the other traits measured on the pregnant cows, elevated body temperature is a clear sign of disease and a common symptom of FT (Burke et al., 2001). In the current study, aRTd increased throughout the trial. In addition, lower hair coat and shedding scores indicate short and slick coats. Gray et al. (2011) found the first month of hair shedding (adaptability score) to be moderately heritable (*h*^2^ = 0.35) and negatively correlated with 205-d adjWW (*r* = −0.58). In this study, HCS and HSS decreased throughout the collection period as expected. However, there was no association between T-Snip rating genotypes and these traits. This lack of associations could indicate that this test is associated with growth traits (aBWd and aBCSd), which may not necessarily be translated/correlated to the other traits analyzed in this study. Lastly, the limited number of animals and difference in endophyte infection rate between locations may have limited the ability to detect associations.

### Effect of T-Snip Rating on Calf Performance

Although no associations between cBW and T-Snip rating genotypes were found, there was a significant genotype-by-environment effect for adjWW, indicating that this genetic test is associated with different weaning performance. As for other traits analyzed, we observed a positive result in UPRS only. Under this environment, in which greater percentages of endophyte infection was observed, animals with greater T-Snip ratings had greater adjWW. This result was expected assuming that the T-Snip is a genetic marker for FT tolerance. This is also in accordance to what was observed originally by Masiero et al. (2016), who also evaluated the growth of calves based on the dam’s T-Snip ratings.

Further studies evaluating this genetic test on spring-born calves would be helpful in gaining a more thorough understanding of how FT effects on weaning weights interact with calving season and T-Snip. At UPRS, cows with greater rating genotypes weaned heavier calves. This result was further supported by our post hoc linear contrast test, which showed that this test, as for aBWd and aBCSd, is additive. This was not observed at BBCFL, further supporting the need of greater endophyte infection percentages in order to observe difference in performance based on T-Snip rating genotypes. However, a statistical association between this genetic test and cBW was not observed, although a numeric trend to the expected direction was observed within UPRS. Overall, these results further support that the T-Snip test may be predictive of calf weaning weight from cows exposed to high levels of toxic fescue during the summer.

## CONCLUSION

This study has evaluated the association between a commercial genetic test for FT with various production traits in pregnant Angus cows when risk of developing FT is greatest. We did not observe association between the T-Snip rating genotypes with most traits evaluated, regardless of the period of time evaluated. However, significant associations were found for major performance traits: cow BW during pregnancy and 205-d adjWW. These associations were observed within one of the two locations used in this study, suggesting that a greater percentage endophyte infection and/or greater concentrations of ergot alkaloids are critical to identify associations between T-Snip rating genotypes and traits of interest affected by FT. In addition, this genetic test was also significantly associated with BCSs of cows. In all these cases, the association between T-Snip genotypes and traits was on the expected direction (i.e., positive) as was indicated by the developers of this genetic test. Finally, this study validated the use of T-Snip for growth traits in Angus cows and calves, and body condition of pregnant cows, while additional studies in different environments throughout the year would be beneficial in validating this commercial genetic test for FT.

## Supplementary Material

txaa181_suppl_Supplementary_Materials
