# Supplementary material for: Evaluation of a commercial genetic test for fescue toxicosis in pregnant Angus beef cattle
Source: Transl Anim Sci. 2020 Oct 1;4(4):txaa181. doi: 10.1093/tas/txaa181 (PMC7724970; doi:10.1093/tas/txaa181)
Supplement: txaa181_suppl_Supplementary_Materials [file txaa181_suppl_supplementary_materials.docx]

**SUPPLEMENTAL MATERIAL**


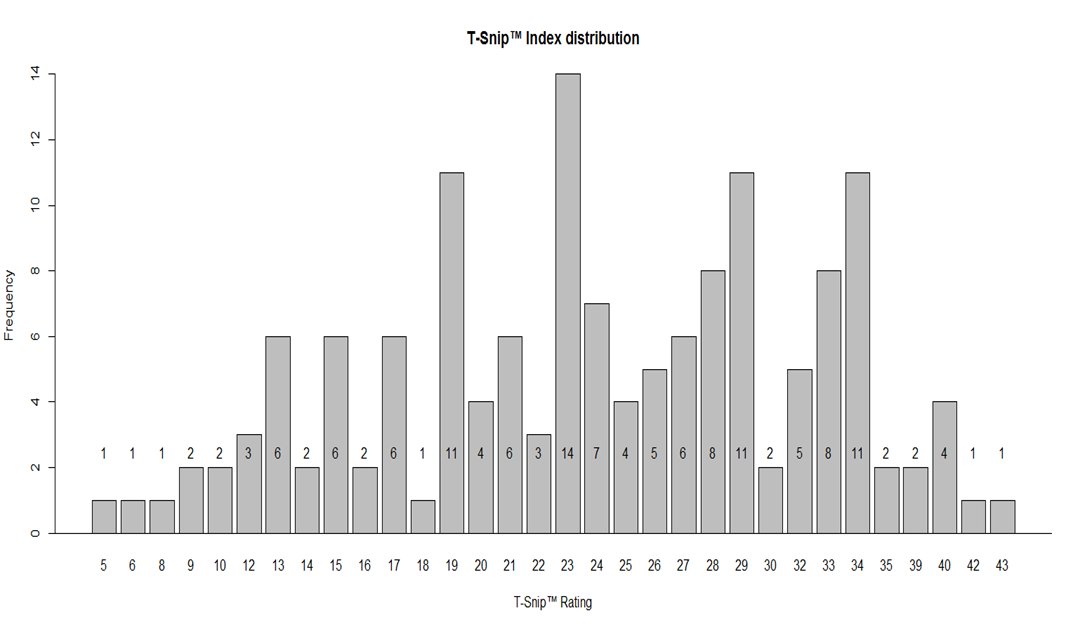


**Figure S1.** **Distribution of T-Snip index genotypes.** Genotype values range from 0 to 50 (x-axis). Each bar represents the frequency of each T-Snip (AgBotanica, LCC, Columbia, MO) genotype, with the frequency on/in each bar.

**Table S1.** Summary statistics of the slope phenotypes used in the final analysis by location^1^ and dataset^2^

|  |  | BBCFL | | | | |  | UPRS | | | | |
| --- | --- | --- | --- | --- | --- | --- | --- | --- | --- | --- | --- | --- |
| Trait^3^ | | n | mean | SD | min | max |  | n | mean | SD | min | max |
| *w1_7* | |  |  |  |  |  |  |  |  |  |  |  |
|  | aBWd, kg/w | 70 | 3.31 | 2.80 | -3.87 | 8.94 |  | 77 | 0.62 | 2.41 | -4.21 | 6.12 |
|  | aBCSd | 70 | 0.00 | 0.04 | -0.09 | 0.11 |  | 77 | 0.05 | 0.08 | -0.12 | 0.27 |
|  | aHCSd | 70 | -0.21 | 0.10 | -0.48 | 0.08 |  | 77 | -0.23 | 0.17 | -0.60 | 0.11 |
|  | aHSSd | 70 | -0.16 | 0.13 | -0.38 | 0.13 |  | 77 | -0.23 | 0.16 | -0.54 | 0.05 |
|  | aRTd, C^o^ | 70 | 0.12 | 0.15 | -0.18 | 0.59 |  | 77 | -0.09 | 0.16 | -0.61 | 0.35 |
| *w7_13* | |  |  |  |  |  |  |  |  |  |  |  |
|  | aBWd, kg/w | 70 | 2.58 | 1.86 | -3.65 | 6.42 |  | 77 | 0.02 | 1.73 | -3.81 | 5.31 |
|  | aBCSd | 70 | 0.10 | 0.05 | 0.01 | 0.23 |  | 77 | 0.06 | 0.06 | -0.13 | 0.20 |
|  | aHCSd | 70 | -0.19 | 0.10 | -0.45 | 0.04 |  | 77 | -0.08 | 0.13 | -0.38 | 0.22 |
|  | aHSSd | 70 | -0.31 | 0.14 | -0.63 | -0.05 |  | 77 | -0.15 | 0.13 | -0.46 | 0.16 |
|  | aRTd, C^o^ | 70 | -0.02 | 0.17 | -0.40 | 0.56 |  | 76 | 0.00 | 0.16 | -0.45 | 0.40 |
| *w0_13* | |  |  |  |  |  |  |  |  |  |  |  |
|  | aBWd, kg/w | 70 | 0.64 | 0.31 | -0.20 | 1.32 |  | 77 | 0.11 | 0.28 | -0.70 | 0.69 |
|  | aBCSd | 70 | 0.05 | 0.02 | 0.01 | 0.10 |  | 77 | 0.05 | 0.03 | -0.03 | 0.11 |
|  | aHCSd | 70 | -0.21 | 0.06 | -0.33 | -0.07 |  | 77 | -0.16 | 0.09 | -0.36 | 0.07 |
|  | aHSSd | 70 | -0.24 | 0.05 | -0.34 | -0.10 |  | 77 | -0.21 | 0.09 | -0.37 | 0.09 |
|  | aRTd, C^o^ | 70 | 0.04 | 0.08 | -0.21 | 0.26 |  | 76 | -0.04 | 0.11 | -0.42 | 0.21 |

^1^BBCFL, Butner Beef Cattle Field Laboratory (Bahama, NC); UPRS, Upper Piedmont Research Station (Reidsville, NC);

^2^Slopes estimated based on 3 time windows: weeks 1 through 13 (w1_13) representing the entirety of the grazing period, weeks 1 through 7 (w1_7), and weeks 7 through 13 (w7_13);

^3^aBWd, average body weight difference; aBCSd, average body condition score difference; aHCSd, average hair coat score difference; aHSSd, average hair shedding score difference; aRTd, average rectal temperature difference.

**Table S2.** Least-squares means for the effect of T-Snip^1^ (TS) rating genotype (1 to 4) by location (L)^2^ analyzing data from weeks 7 to 13

|  | BBCFL | | | |  | UPRS | | | |  | *P-*value | | | |
| --- | --- | --- | --- | --- | --- | --- | --- | --- | --- | --- | --- | --- | --- | --- |
| Trait^3^ | 1 | 2 | 3 | 4 |  | 1 | 2 | 3 | 4 | pSEM^4^ | TS*L | TS | L |  |
| aBWd, kg/w | 2.14^bc^ | 3.15^ab^ | 2.44^b^ | 4.05^a^ |  | -0.75^e^ | -0.01^de^ | -0.26^de^ | 0.71^cd^ | 0.40 | 0.860 | 0.032 | 0.060 |  |
| aBCSd | 0.123^a^ | 0.110^a^ | 0.109^a^ | 0.092^ab^ |  | 0.039^c^ | 0.051^bc^ | 0.086^ab^ | 0.066^abc^ | 0.015 | 0.165 | 0.393 | <0.01 |  |
| aHCSd | -0.151^ab^ | -0.199^b^ | -0.190^b^ | -0.151^ab^ |  | -0.143^ab^ | -0.121^a^ | -0.89^a^ | -0.125^ab^ | 0.028 | 0.452 | 0.792 | 0.060 |  |
| aHSSd | -0.294^b^ | -0.324^b^ | -0.308^b^ | -0.324^b^ |  | -0.209^ab^ | -0.187^ab^ | -0.164^a^ | -0.180^ab^ | 0.032 | 0.879 | 0.866 | <0.01 |  |
| aRTd, C^o^ | -0.117 | -0.034 | -0.064 | -0.038 |  | -0.035 | -0.069 | -0.001 | -0.036 | 0.041 | 0.400 | 0.783 | 0.512 |  |

^a-e^Least-squares means lacking common superscripts are statistically different (*P* < 0.1). Difference in means were included for all analyses, regardless of the significance of effects, for completeness;

^1^AgBotanica, LCC, Columbia, MO;

^2^BBCFL, Butner Beef Cattle Field Laboratory (Bahama, NC); UPRS, Upper Piedmont Research Station (Reidsville, NC);

^3^aBWd, average body weight difference; aBCSd, average body condition score difference; aHCSd, average hair coat score difference; aHSSd, average hair shedding score difference; aRTd, average rectal temperature difference; cBW, calf birth weight; adjWW, 205-d adjusted calf weaning weight;

^4^Pooled SEM, calculated as the weighted average of SEM at each location-genotype combination.

**Table S3.** Least-squares means for the effect of T-Snip^1^ (TS) rating genotype (1 to 4) by location (L)^2^ analyzing data from weeks 1 to 13

|  | BBCFL | | | |  | UPRS | | | |  | | *P-*value | | |
| --- | --- | --- | --- | --- | --- | --- | --- | --- | --- | --- | --- | --- | --- | --- |
| Trait^3^ | 1 | 2 | 3 | 4 |  | 1 | 2 | 3 | 4 | pSEM^4^ | TS*L | | TS | L |
| aBWd, kg/w | 0.779^a^ | 0.647^a^ | 0.648^a^ | 0.766^a^ |  | -0.080^d^ | 0.067^c^ | 0.086^c^ | 0.325^b^ | 0.067 | 0.152 | | 0.329 | <0.01 |
| aBCSd | 0.076^a^ | 0.056^ab^ | 0.053^ab^ | 0.058^ab^ |  | 0.036^b^ | 0.043^b^ | 0.054^ab^ | 0.075^a^ | 0.007 | 0.819 | | 0.126 | <0.01 |
| aHCSd | -0.200^ab^ | -0.207^b^ | -0.192^ab^ | -0.192^ab^ |  | -0.205^ab^ | -0.171^a^ | -0.193^ab^ | -0.206^ab^ | 0.014 | 0.369 | | 0.492 | 0.337 |
| aHSSd | -0.267^bc^ | -0.237^bc^ | -0.247^bc^ | -0.263^bc^ |  | -0.237^bc^ | -0.206^a^ | -0.237^b^ | -0.289^c^ | 0.015 | 0.277 | | 0.754 | 0.093 |
| aRTd, C^o^ | 0.005 | 0.027 | 0.014 | -0.003 |  | 0.042 | 0.023 | 0.011 | 0.041 | 0.016 | 0.545 | | 0.321 | <0.01 |

^a-e^Least-squares means lacking common superscripts are statistically different (*P* < 0.1). Difference in means were included for all analyses, regardless of the significance of effects, for completeness;

^1^AgBotanica, LCC, Columbia, MO;

^2^BBCFL, Butner Beef Cattle Field Laboratory (Bahama, NC); UPRS, Upper Piedmont Research Station (Reidsville, NC);

^3^aBWd, average body weight difference; aBCSd, average body condition score difference; aHCSd, average hair coat score difference; aHSSd, average hair shedding score difference; aRTd, average rectal temperature difference; cBW, calf birth weight; adjWW, 205-d adjusted calf weaning weight;

^4^Pooled SEM, calculated as the weighted average of SEM at each location-genotype combination.
